# *Bacillus subtilis* Bacteremia from Gastrointestinal Perforation after Natto Ingestion, Japan

**DOI:** 10.3201/eid2910.230084

**Published:** 2023-10

**Authors:** Takehiro Hashimoto, Takaaki Yahiro, Sakirul Khan, Kazunori Kimitsuki, Kazufumi Hiramatsu, Akira Nishizono

**Affiliations:** Oita University Hospital, Oita, Japan (T. Hashimoto, K. Hiramatsu);; Oita University Faculty of Medicine, Oita (T. Hashimoto, T. Yahiro, S. Khan, K. Kimitsuki, A. Nishizono);; Research Center for Global and Local Infectious Diseases, Oita (K. Hiramatsu, A. Nishizono)

**Keywords:** *Bacillus subtilis*, bacteria, food safety, enteric infections, bacteremia, gastrointestinal perforation, natto, Japan

## Abstract

We report a case of *Bacillus subtilis* variant *natto* bacteremia from a gastrointestinal perforation in a patient who ingested natto. Genotypic methods showed the bacteria in a blood sample and the ingested natto were the same strains. Older or immunocompromised patients could be at risk for bacteremia from ingesting natto.

*Bacillus subtilis* is a gram-positive, rod-shaped, spore-forming bacterium with low pathogenicity (*1*). *B. subtilis* isolated from clinical specimens is sometimes considered a contaminant (*2*). However, a few cases of bacteremia caused by *B. subtilis* have been reported in Japan (*3*–*6*). We report a case of *B. subtilis* variant *natto* bacteremia and peritonitis caused by ingestion of natto, a traditional fermented food in Japan that is prepared by adding *B. subtilis* var. *natto* culture to soybeans and fermenting them.

A 65-year-old man with metastatic colorectal cancer was admitted to Oita University Hospital (Oita, Japan) with fever and perianal pain. Approximately 2 months before admission, he began chemotherapy with bevacizumab and modified oxaliplatin plus leucovorin plus 5-fluorouracilm (FOLFOX6). Six days before admission, on day 4 after the third course of chemotherapy, he experienced perianal pain. He had a history of diabetes mellitus and a custom of eating natto. 

The patient’s body temperature was 37.6°C, blood pressure was 132/84 mm Hg, and heart rate was 70 beats/min. A physical examination revealed lower abdominal pain. Laboratory tests revealed an elevated leukocyte count (15,150 cells/μL), elevated C-reactive protein level (20.2 mg/dL), and elevated procalcitonin level (0.55 ng/mL). Contrast-enhanced abdominal computed tomography showed free air in the perisigmoid colon, an intraabdominal abscess, and slight ascites. Peritonitis caused by sigmoid colon perforation was diagnosed, and a transverse colostomy and drainage were performed. After blood and pus cultures were collected, a course of intravenous meropenem (0.5 g every 8 h) was initiated.

On day 3, matrix-assisted laser desorption/ionization time-of-flight (MALDI-TOF) mass spectrometry (Bruker Daltonics, https://www.bruker.com) of the blood culture revealed *B. subtilis* (MALDI-TOF score 2.224), and the pus culture revealed polymicrobial bacteria, including *B. subtilis*. We performed antimicrobial resistance testing on a dry plate (Eiken Chemical Co., https://www.eiken.co.jp) by using the broth microdilution method and then analyzed images by using a Koden IA40MIC-i (Koden, https://koden.jp). MICs were as follows: cefazolin, <0.25 µg/mL; cefotiam, 0.5 µg/mL; meropenem, <0.25 µg/mL; clindamycin, 0.5 µg/mL; levofloxacin <0.25 µg/mL; minocycline, <0.25 µg/mL; and vancomycin, <0.5 µg/mL. Meropenem was administered for a total of 10 days. On day 36, additional computed tomography–guided percutaneous drainage was performed, and on day 66, the patient was discharged from the hospital after rehabilitation. 

We assessed whether blood and pus culture isolates and the isolate from natto the patient consumed (brand A) were the same strain, by using pulsed-field gel electrophoresis (PFGE), as previously described (*7*). To verify *B. subtilis* var. *natto*, we tested 2 brands (brands B and C) besides brand A that the patient reported consuming. PFGE revealed that the isolates detected from the blood and pus cultures were the same as the cultures of natto brand A that the patient consumed. Moreover, those isolates were the same as isolates from natto brand C (Figure), suggesting that the blood and pus culture isolates were *B. subtilis* var. *natto*.

**Figure:**
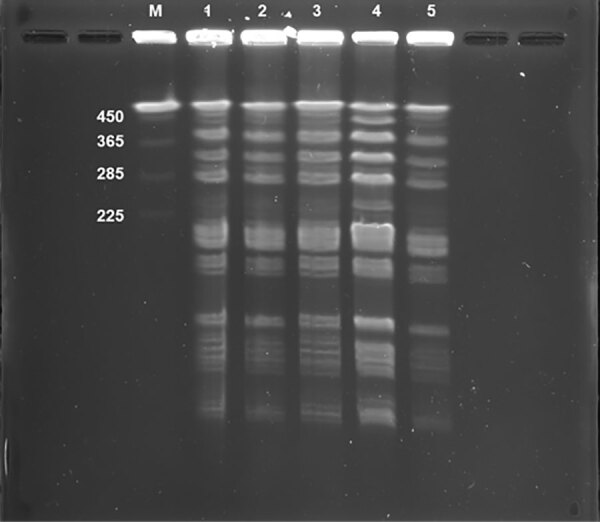
Pulsed-field gel electrophoresis patterns of restricted chromosomal DNA from *Bacillus subtilis* variant *natto* isolated from a case of bacteremia from gastrointestinal perforation after natto ingestion, Japan. *B. subtilis* strains were digested in Sfil enzyme. Lane M, CHEF DNA size marker (Bio-Rad, https://www.bio-rad.com) of *Saccharomyces cerevisiae*; lane 1, sample from blood; lane 2, sample from pus; lane 3, sample from natto brand A; lane 4, sample from natto brand B; lane 5, sample from natto brand C. Numbers at left indicate kilobases.

Other than *B. subtilis* var. *natto, B. subtilis* has multiple other subspecies: *B. subtilis* subsp. *subtilis*, *B. subtilis* subsp. *spizizenii*, *B. subtilis* subsp. *inaquosrum*, and *B. subtilis* subsp. *stercoris*. Distinguishing between those species by 16S rRNA gene sequencing and MALDI-TOF mass spectrometry can be difficult (*8*). The most common known portal of entry for *B. subtilis* bacteria is the gastrointestinal tract, but often the site of entry is unknown, and bacteremia that has a gastrointestinal tract source is presumed to be related to natto ingestion (*3*–*6*). However, *B. subtilis* var. *natto* was identified in only 2 prior cases, 1 that analyzed the draft whole-genome of each *B. subtilis* strain using next-generation sequencing (*6*) and 1 that used the biotin gene and the biotin requirement test (*5*). In our case, PFGE analysis revealed that the patient’s isolate was *B. subtilis* var. *natto* that matched the natto brand he consumed. Although *B. subtilis* bacteremia has been reported only in Japan thus far, the popularity of Japanese cuisine is increasing worldwide (*9*). Therefore, clinicians outside Japan should also be aware of *B. subtilis* bacteremia caused by natto consumption.

PFGE analysis revealed that the natto of brands A and B contained different bacterial strains. Many brands of natto are sold in Japan. Each brand uses different soybean cultivars, processing conditions (soaking, steaming, and fermentation), and *B. subtilis* var. *natto* strains (*10*). Therefore, a history of natto consumption alone might not be associated with the cause of *B. subtilis* bacteremia because eating natto is not uncommon among the population of Japan.

In conclusion, we report a case of *B. subtilis* bacteremia and secondary peritonitis resulting from gastrointestinal perforation in a patient who ingested natto. Our case and others in the literature indicate that older or immunocompromised patients who consume natto are at risk for serious infection from natto (*4*,*6*). Clinicians should advise patients in these risk groups to avoid eating natto or food products containing *B. subtilis* bacteria.
